# EphA5 Expression Predicts Better Survival Despite an Association with Proliferative Activity in Endometrial Cancer

**DOI:** 10.3390/jcm14155360

**Published:** 2025-07-29

**Authors:** Shy-Yau Ang, Ching-Yu Shih, Hua Ho, Yen-Lin Chen, Jen-Tang Sun, Chiao-Yin Cheng

**Affiliations:** 1 Department of Emergency Medicine, Far Eastern Memorial Hospital, New Taipei 220, Taiwan; fynestsy@gmail.com (S.-Y.A.); h790317@gmail.com (H.H.); 2 Center for Precision Medicine and Genomics, Tri-Service General Hospital, National Defense Medical University, Taipei 114, Taiwan; c588011124@gmail.com; 3 Center for Precision Medicine and Genomics, Department of Pathology, Tri-Service General Hospital, National Defense Medical University, Taipei 114, Taiwan; yenlinchen12742@mail.ndmctsgh.edu.tw

**Keywords:** endometrial cancer, EphA5, Ki-67, metabolic stress, overall survival, pAMPK, tumor biomarker, tumor cell proliferation

## Abstract

**Background/Objectives**: Eph receptor A5 (EphA5) is a receptor tyrosine kinase that is implicated in multiple malignancies, although its role in endometrial cancer (EC) remains unclear. The aim of this study was to investigate the clinicopathological significance of EphA5 expression in EC and explore its association with proliferative and metabolic markers. **Methods**: We retrospectively analyzed 75 EC tissue samples from treatment-naïve patients by using immunohistochemistry and H-score quantification. Associations between EphA5 expression and clinicopathological parameters were assessed through logistic regression analysis. Kaplan–Meier analysis was used to evaluate survival outcomes. Correlation analysis, stratified according to cancer stage, was used to explore biomarker interactions. **Results**: High EphA5 expression levels were significantly associated with elevated Ki-67 expression (adjusted odds ratio (aOR): 1.08 per 1-point H-score increase, *p* = 0.024) and decreased pAMPK expression (aOR: 0.89 per 1-point H-score increase, *p* = 0.024), indicating its involvement in proliferative and metabolic pathways. Paradoxically, patients with high EphA5 levels had significantly better overall survival probabilities (H-score > 105, log-rank *p* = 0.007). Stage-specific analyses suggested that EphA5 levels correlated with proliferation in early-stage disease and epithelial–mesenchymal transition in advanced stages. **Conclusions**: EphA5 may act as a context-dependent biomarker in EC. Despite its positive correlation with proliferation and negative association with metabolic stress signaling, high EphA5 expression levels were predictive of a favorable prognosis.

## 1. Introduction

Endometrial cancer (EC) is the most common gynecologic malignancy in high-income countries, with both incidence and mortality continuing to rise globally [1]. It accounts for approximately 6% of all female cancers in the United States, with an estimated 320,000 new cases and 76,000 deaths annually worldwide [2]. Major risk factors include obesity, metabolic syndrome, type 2 diabetes, unopposed estrogen exposure, late menopause, and genetic predispositions such as Lynch syndrome [2]. EC is commonly classified into two types: types I (estrogen-dependent, better prognosis) and II (non-estrogen-dependent, poorer prognosis). Endometrial stromal tumors, a rare subtype of uterine mesenchymal neoplasms, have an incidence of about 0.3 per 100,000 women and primarily affect peri- or postmenopausal individuals [2]. Low-grade endometrial stromal sarcomas (LG-ESSs) progress slowly and are often diagnosed in the early stages with favorable outcomes, whereas high-grade endometrial stromal sarcomas (HG-ESSs) and undifferentiated uterine sarcomas (UUSs) are aggressive, with poor prognoses. Treatment for LG-ESS includes hysterectomy with bilateral salpingo-oophorectomy and possible hormonal therapy for advanced or recurrent cases, whereas HG-ESS and UUS often require multimodal treatment, including surgery and chemotherapy. Although endometriosis is not directly linked to EC, it shares overlapping estrogen-related pathways and risk factors and remains a prevalent condition among women of reproductive age [3].

Several biomarkers have been identified as important prognostic indicators in EC, contributing to risk stratification and personalized treatment approaches. The Cancer Genome Atlas molecular classification divides EC into four subtypes—POLE ultramutated, mismatch repair-deficient/microsatellite instability-high, copy number low/no specific molecular profile, and copy number high/p53 abnormal—with *POLE* mutations predicting excellent outcomes and p53 abnormalities indicating a poor prognosis [4,5]. Human epididymis protein 4 has shown promise as a biomarker for disease progression and survival, with higher serum levels associated with more advanced stages and worse overall survival [6,7]. Circular RNAs, such as circWDR26 and circTNFRSF21, have been implicated in EC tumorigenesis and are associated with unfavorable prognoses owing to their roles in cell proliferation and migration [8]. Additionally, metabolomic biomarkers—altered levels of metabolites such as lactate, glutamate, and phosphocholine—have been linked to disease recurrence and aggressiveness [9].

Eph receptor A5 (EphA5) is a member of the Eph family of receptor tyrosine kinases, which play critical roles in cell positioning, migration, and tissue architecture during development. The *EPHA5* gene was officially designated under a unified Eph receptor nomenclature system in 1997 to standardize research across this receptor family [10]. Although EphA5 is predominantly studied in the context of neural development, recent evidence suggests that it may also function as an oncogenic driver in certain cancers. Notably, in follicular thyroid carcinoma, EphA5 reportedly promotes tumor cell proliferation and suppresses apoptosis via activation of the STAT3 signaling pathway, implicating it in tumor progression and a poor prognosis [11]. Although direct evidence in EC remains limited, the oncogenic mechanisms by which EphA5 is involved in other malignancies suggest that it may serve as a prognostic biomarker or therapeutic target in EC. Further investigations are needed to clarify its role in the molecular landscape of endometrial tumorigenesis.

EphA5 has been increasingly recognized as a prognostic biomarker in various cancers, with its role varying depending on tumor type. In non-small cell lung cancer, *EPHA5* mutations are linked to poorer survival among patients treated with atezolizumab, suggesting a negative prognostic impact in immunotherapeutic contexts [12]. Conversely, in lung adenocarcinoma, *EPHA5* mutations correlate with enhanced immune infiltration and better immunotherapeutic responses [13]. In esophageal adenocarcinoma, a gene signature that includes *EPHA5* mutations predicted an improved response to neoadjuvant chemoradiotherapy in one study [14], whereas in colorectal cancer, reduced EphA5 expression is associated with lymph node metastasis, an advanced stage, and a poor prognosis [15]. Similarly, in prostate cancer, EphA5 downregulation via hypermethylation is linked to a higher tumor grade and invasiveness [16]. In ovarian serous carcinoma, positive EphA5 expression is associated with longer survival [17], and in esophageal squamous cell carcinoma, EphA5 knockdown promotes proliferation and invasion through the Wnt/β-catenin pathway [18]. Moreover, in bladder cancer, lower EphA5 levels paradoxically indicate better overall survival [19], whereas in glioma, elevated EphA5 expression correlates with tumor progression [20]. Those results underscore the context-dependent prognostic relevance of EphA5, functioning as either a tumor suppressor or a promoter across different malignancies.

The aim of this study was to investigate the prognostic significance of EphA5 expression in EC. Given the established role of EphA5 in tumor progression and immune modulation across various malignancies, including lung, colorectal, and ovarian cancers, it may serve as a context-dependent biomarker of patient outcomes. We evaluated the association between EphA5 expression levels and clinicopathological characteristics as well as survival outcomes in patients with EC and explored the correlation of EphA5 expression with the expression of other commonly studied biomarkers. We wanted to determine whether EphA5 can serve as a prognostic biomarker, to elucidate potential pathways through which it may affect disease progression, and to provide insights that may inform risk stratification and therapeutic decision-making in EC. We discovered that, although EphA5 expression was linked to proliferative signaling and metabolic regulation, it was linked to better overall survival.

## 2. Materials and Methods

**Figure 1:**
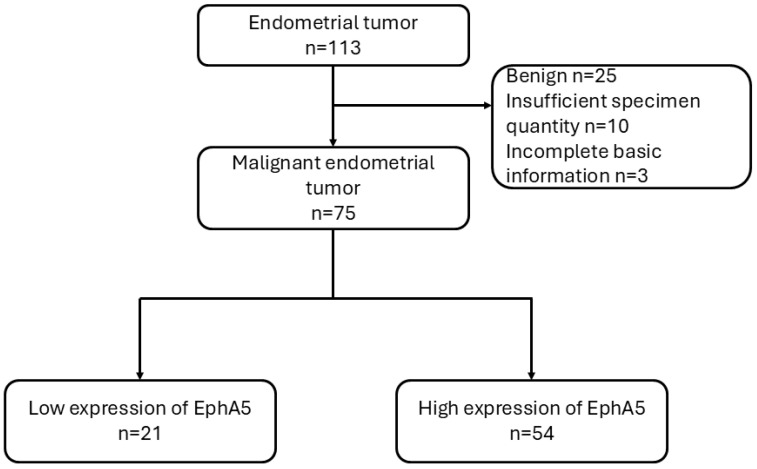
Flowchart of case selection. A total of 113 endometrial tumor specimens were collected. After excluding benign tumors and cases with insufficient samples or incomplete data (n = 38), 75 malignant cases were included and classified into low (n = 21) and high (n = 54) EphA5 expression groups.

A total of 113 endometrial tumor specimens were collected from patients treated between 2001 and 2017. After excluding benign tumors (n = 25), cases with insufficient specimen quantity (n = 10), and those with incomplete basic clinical information (n = 3), 75 malignant endometrial tumor cases were included for analysis. These cases were further classified into two groups based on EphA5 expression levels as determined by immunohistochemistry: low expression (n = 21) and high expression (n = 54). Patient outcomes were subsequently analyzed, with a maximum follow-up period of up to 17 years from the time of diagnosis (Figure 1). All procedures were approved by the Institutional Review Board of Cardinal Tien Hospital (approval no.: CTH-106-2-5-042). Clinical data, including patient age, tumor size, histological grade, International Federation of Gynecology and Obstetrics (FIGO) stage, and survival outcomes, were retrieved from medical records.

For each case, a tumor core (2 mm in diameter) was selected and integrated into a tissue microarray. The microarray blocks were embedded in paraffin and cut into sections of 0.3 µm in thickness. Sections were mounted onto adhesive-coated slides and baked at 65 °C for one hour to ensure tissue adherence. They were deparaffinized and rehydrated using two 10-min xylene immersions, followed by dehydration via a graded ethanol series (100%, 95%, and 75%) and a final rinse in distilled water.

Immunohistochemical staining was performed using the automated Ventana BenchMark XT system (Ventana Medical Systems, Tucson, AZ, USA) under standardized conditions. After rehydration, tissue sections were washed with phosphate-buffered saline and subjected to heat-induced epitope retrieval in ethylenediaminetetraacetic acid buffer (pH 8.0) for 24 min, optimized for each antibody. Slides were incubated with primary antibodies at 37 °C for one hour. Immunodetection was achieved using diaminobenzidine as the chromogen, and hematoxylin was used for nuclear counterstaining.

Based on our team’s previous research [21,22], we selected the following primary antibodies: pAMPK (1:100; Cell Signaling Technology, Danvers, MA, USA; lot no.: 2535), pStat3 (1:50; Abcam, Cambridge, UK; lot no.: ab76315), pAkt (1:50; GeneTex, Irvine, CA, USA; lot no.: GTX11901), pErk (1:200; R&D Systems, Minneapolis, MN, USA; lot no.: AF1018), N-cadherin (1:100; Abcam; lot no.: ab76011), E-cadherin (1:100; Abcam; lot no.: ab40772), CD31 (1:500; Abbiotec, Escondido, CA, USA; lot no.: 250590), fibronectin (1:50; Santa Cruz Biotechnology, Dallas, TX, USA; lot no.: SC-8422), caspase-3 (1:100; Cell Signaling Technology; lot no.: 9664), Ki-67 (1:100; BioLegend, San Diego, CA, USA; lot no.: 350503), and EphA5 (1:200; Thermo Fisher Scientific, Waltham, MA, USA; lot no.: PA5-14581). All antibodies were titrated to achieve optimal signal-to-noise ratios.

**Figure 2:**
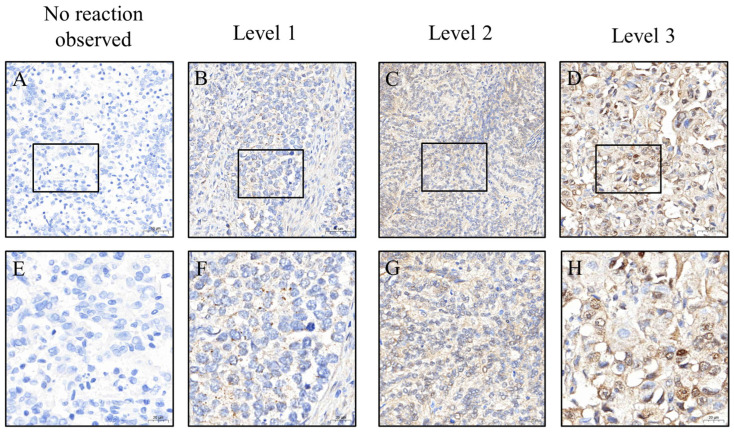
Immunohistochemical staining of EphA5 in EC tissues, illustrating different expression levels. Panels (**A**–**D**) are shown at 200× magnification, with scale bars of 50 μm, whereas panels (**E**–**H**) are shown at 400× magnification, with scale bars of 20 μm. The box indicates the magnified region. EC, endometrial cancer; EphA5, Eph receptor 5.

**Figure 3:**
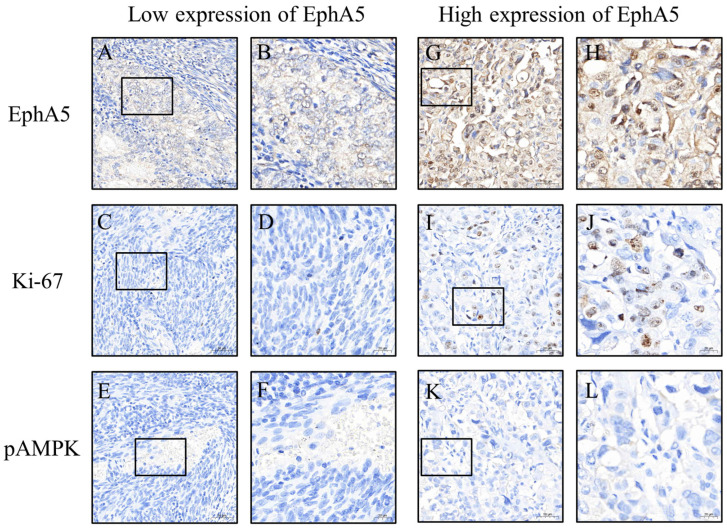
Representative images of Ki-67 and pAMPK expression levels in EC tissues with low and high EphA5 expression levels. Panels (**A**–**F**) correspond to low EphA5 expression levels, whereas panels (**G**–**L**) represent high EphA5 expression levels. Panels (**A**,**C**,**E**,**G**,**I**,**K**) are shown at 200× magnification, with scale bars of 50 μm, whereas panels (**B**,**D**,**F**,**H**,**J**,**L**) are shown at 400× magnification, with scale bars of 20 μm. The box indicates the magnified region.

To ensure consistent and objective immunohistochemical evaluation, all tissue sections were stained using an automated platform operated by technicians blinded to clinical information. Whole-slide images were captured at 200× magnification using a 3DHISTECH scanner (Budapest, Hungary). Regions of interest (ROIs) were identified and H-scores were initially calculated using PatternQuant (3DHISTECH, Budapest, Hungary; version 2.4). This software quantifies immunostaining by multiplying the staining intensity (0 = none, 1 = weak, 2 = moderate, and 3 = strong) by the percentage of positively stained tumor area (0–100%), resulting in a semi-quantitative score ranging from 0 to 300 (Figure 2 and Figure 3). To validate these results, the percentage of positive staining and intensity grading were independently reassessed using ImageJ software (version 1.54f; NIH, Bethesda, MD, USA). All automated and manual assessments were reviewed by Dr. Yen-Lin Chen, a board-certified pathologist, who verified the accuracy and consistency of the final dataset. This dual-layered approach ensured robust and reproducible quantification of biomarker expression.

For EphA5, an H-score cutoff of 105 was applied to stratify patients into high- and low-expression groups. This threshold was selected based on the score distribution and its clinical relevance to survival outcomes. Kaplan–Meier survival curves were generated to evaluate overall survival, and differences between groups were assessed using the log-rank test. Age, tumor size, and the H-scores of the markers were treated as continuous variables, while sex, tumor differentiation, and FIGO stage were treated as categorical variables. We first used the Kolmogorov–Smirnov test to assess whether the data conformed to a normal distribution. None of the continuous variables in this study were normally distributed; therefore, we used the Mann–Whitney U test to determine whether there were significant differences. For categorical variables, we used the chi-square test for analysis correlations between EphA5 expression, and other variables were analyzed using Pearson’s correlation. Logistic regression models (both univariate and multivariable) were used to identify independent predictors of a high EphA5 expression level. All statistical analyses were conducted using IBM SPSS Statistics version 26.0 (IBM Corp., Armonk, NY, USA), with *p*-values < 0.05 considered statistically significant. Kaplan–Meier survival plots were generated with the assistance of the lifelines module in Python 3.13.3 (Wilmington, DE, USA).

## 3. Results

We first investigated the association between EphA5 expression levels and clinicopathological characteristics in patients with EC. The median ages of the low- (n = 21) and high-expression (n = 54) groups were 57.0 years (quartiles 1–3: 52.0–63.5) and 54.0 years (48.0–60.0), respectively, not a significant difference (*p* = 0.096). Histological grading revealed a significantly higher proportion of poorly differentiated tumors in the low-expression group compared with that in the high-expression group (38% vs. 11%, *p* = 0.014). Tumor size did not significantly differ between the groups (4.0 cm vs. 2.8 cm, *p* = 0.282). However, FIGO clinical staging revealed a significantly larger proportion of advanced-stage (stage III) cases in the low-expression group (29% vs. 4%, *p* = 0.006). Taken together, a low EphA5 expression level was significantly associated with poor differentiation and advanced disease stage, suggesting its potential as a biomarker for an unfavorable prognosis (Table 1).

To further explore the biological implications of EphA5 expression, we compared the profiles of other biomarkers between the high- and low-expression groups. A high EphA5 expression level was significantly associated with elevated levels of N-cadherin (*p* = 0.003), pAkt (*p* = 0.003), and pStat3 (*p* = 0.001), indicating potential involvement in epithelial–mesenchymal transition, pro-survival signaling, and inflammatory responses. Conversely, the expression level pAMPK, a key regulator of energy homeostasis, was significantly lower in the high-expression group (*p* = 0.004), suggesting the suppression of tumor-suppressive metabolic pathways. Ki-67 expression might also have been higher in the high-expression group (*p* = 0.050), indicating that proliferative activity may be enhanced in tumors with high EphA5 expression levels. Although the other biomarkers (caspase-3, CD31, E-cadherin, fibronectin, and pErk) did not significantly differ between the groups, their distribution patterns may warrant further investigation (Table 2).

We subsequently performed logistic regression analyses to identify factors associated with a high EphA5 expression level. A higher Ki-67 expression level was independently associated with a higher EphA5 expression level (adjusted odds ratio [aOR]: 1.08 per 1-point H-score increase; 95% confidence interval [CI]: 1.01–1.16; *p* = 0.024), reinforcing the link between EphA5 expression and cellular proliferation. Conversely, a higher pAMPK expression level was independently associated with a lower EphA5 expression level (aOR: 0.89 per 1-point H-score increase; 95% CI: 0.81–0.98; and *p* = 0.024), suggesting an inverse relationship between EphA5 expression and energy metabolism regulation. Additionally, a low tumor grade might have been inversely associated with the EphA5 expression level (aOR: 0.06; 95% CI: 0.00–1.05; and *p* = 0.054), and a FIGO stage III classification was independently associated with a lower EphA5 expression level (aOR: 0.06; 95% CI: 0.00–0.95; *p* = 0.046). The significance of other markers was not retained upon multivariable analysis (Table 3).

**Figure 4:**
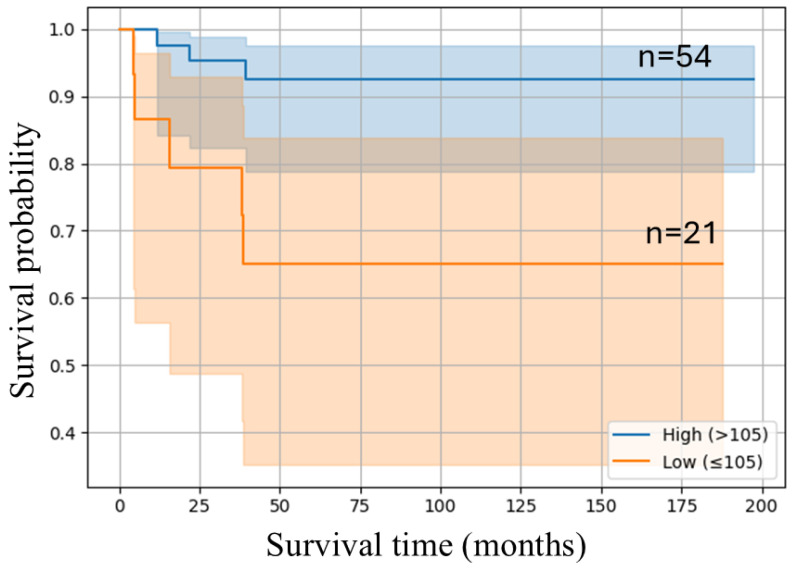
Kaplan–Meier survival curves of overall survival between patients with low EphA5 expression levels and those with high expression levels. The log-rank test revealed a statistically significant difference between them (*p* = 0.007). The shaded regions surrounding the curves represent 95% confidence intervals.

We performed Kaplan–Meier survival analysis to assess the prognostic relevance of EphA5 expression. As shown in Figure 4, patients with a high EphA5 expression level exhibited significantly better overall survival than those with a lower expression level. The high-expression group maintained a higher survival probability throughout the follow-up period, while the low-expression group experienced a steep decline, particularly within the first 50 months. The 95% CIs were narrower in the high-expression group, indicating more consistent outcomes. These results suggest that elevated EphA5 expression levels are associated with an improved prognosis in EC (log-rank test, *p* = 0.007).

To evaluate the molecular correlates of EphA5 expression across disease stages, Pearson correlation analyses were performed within subgroups stratified according to cancer stage (Table 4). In patients with stage I EC (n = 61), the EphA5 expression level was negatively correlated with tumor grade (r = −0.262, *p* = 0.041) and positively correlated with the Ki-67 expression level (r = 0.255, *p* = 0.047), supporting its association with both favorable differentiation and increased proliferation. A strong inverse correlation was also observed with the pAMPK expression level (r = −0.447, *p* < 0.001), suggesting potential involvement in metabolic regulation.

In contrast, patients with stage II EC (n = 6) exhibited no significant correlations; however, moderate, nonsignificant associations were observed with pStat3 (r = 0.672, *p* = 0.144) and pAkt (r = 0.480, *p* = 0.335) expression levels, which may warrant further investigation in larger cohorts. Among patients with stage III disease (n = 8), strong positive correlations were observed between EphA5 and both Ki-67 (r = 0.909, *p* = 0.002) and N-cadherin (r = 0.882, *p* = 0.004) expression levels. These results suggest that EphA5 may play distinct biological roles across cancer stages, particularly in relation to proliferation, differentiation, and metabolic signaling.

Finally, we conducted a subgroup analysis to compare the median expression levels and interquartile ranges (IQRs) of various tumor-related biomarkers between low and high EphA5 expression groups across different FIGO stages (I–III), along with the corresponding *p*-values. In FIGO stage I, significant differences were observed in pAkt (*p* = 0.006), pStat3 (*p* = 0.012), and pAMPK (*p* = 0.001), where high EphA5 expression was associated with elevated pAkt and pStat3 levels but lower pAMPK expression. Additionally, Ki-67 and N-cadherin showed borderline significance (*p* ≈ 0.05–0.07), suggesting potential differential expression. In contrast, most biomarkers in FIGO stages II and III did not show statistically significant differences between EphA5 expression groups, which may be attributed to limited sample sizes or tumor heterogeneity. These findings suggest that EphA5 may play a regulatory role in early tumor progression, particularly through interactions with key signaling molecules such as pAMPK, pAkt, and pStat3, warranting further mechanistic investigation (Table S1). Table S2 presents univariable and multivariable regression analyses restricted to stage I cases (n = 61). A low tumor grade and low pAMPK expression level were independently associated with a high EphA5 expression level in both models, supporting results from the full cohort analysis. Table S3 summarizes the univariable logistic regression results for stage II (n = 6) and III (n = 8) cases. Owing to the limited sample sizes in these subgroups, multivariable regression was not performed to avoid statistical overfitting. No significant associations were identified.

## 4. Discussion

In this study, we identified significant associations between EphA5 expression and key clinicopathological and molecular features of EC. Multivariable logistic regression analysis revealed that a high Ki-67 expression level (aOR: 1.08, *p* = 0.024) was an independent predictor of an elevated EphA5 level, supporting its link to proliferative activity. Conversely, increased pAMPK expression levels were associated with reduced EphA5 expression levels (aOR: 0.89, *p* = 0.024), suggesting a role of suppression of energy metabolism in EphA5-mediated oncogenic pathways. Additionally, a low tumor grade and early FIGO stage seemed inversely related to the EphA5 expression level, although only the relationship with a FIGO stage III was significant (aOR: 0.06, *p* = 0.046). Kaplan–Meier survival analysis further demonstrated that patients with a high EphA5 expression level (H-score > 105) exhibited significantly higher overall survival compared with those with low expression levels, indicating its potential as a favorable prognostic biomarker. Stratified correlation analysis across cancer stages revealed stage-specific biological roles of EphA5. In stage I tumors, the EphA5 expression level was positively correlated with the Ki-67 expression level and inversely with the tumor grade and pAMPK expression level. In stage III tumors, the EphA5 expression level strongly correlated with both Ki-67 and N-cadherin expression levels. These correlations suggest the involvement of EphA5 in proliferative signaling and epithelial–mesenchymal transition in advanced disease.

Emerging evidence suggests that EPHA5 may serve as a context-dependent regulator in multiple malignancies. In lung adenocarcinoma, EPHA5 expression is positively correlated with EGFR mutation status and lymph node metastasis, implicating its potential role in tumor progression and molecular classification [23]. In cervical cancer, integrative transcriptomic meta-analyses identified EPHA5 among a panel of dysregulated genes involved in tumorigenesis. Specifically, EPHA5 and related ephrin signaling molecules (e.g., EPHA4, EPHB2, and EDNRA) were shown to participate in oncogenic networks modulating cell proliferation, migration, and immune response regulation [24]. These findings were derived from multi-platform datasets, including mRNA, microRNA, and proteomic profiles, and validated across independent cohorts. The implication of EPHA5 in cervical carcinogenesis expands its relevance beyond lung cancer and supports its potential as a cross-tumor biomarker. Moreover, antibody-based therapeutic strategies targeting EPH receptors are currently under development and may provide a translational avenue for future therapeutic applications [25].

Ki-67 is a well-established nuclear protein marker of cellular proliferation, expressed during all active phases of the cell cycle (G1, S, G2, and M) but absent in quiescent cells (G0). In EC, elevated Ki-67 expression is strongly associated with higher tumor grade, advanced FIGO stage, and aggressive pathological features; thus, it is a valuable indicator of tumor biology and prognosis. Several studies [26,27] demonstrated that high Ki-67 levels correlate with poor disease-free and overall survival, particularly in patients with high-grade tumors and those who exhibit myometrial invasion or lymphovascular involvement. Moreover, Ki-67 has been incorporated into risk stratification models alongside other markers, such as ER, PR, p53, and L1CAM, and it may help to guide treatment decisions. Importantly, even in low-grade (grades 1–2) EC, elevated Ki-67 expression has been linked to an increased likelihood of aggressive behavior in prior studies [26] and in our present study. Additionally, Ki-67 expression levels vary among molecular EC subtypes, being highest in p53-abnormal tumors, which typically have the worst prognosis [28]. Our study further supports those observations, showing that high Ki-67 expression is significantly associated with advanced-stage disease and worse clinical outcomes. Notably, this is the first report to show that elevated EphA5 expression, although positively correlated with increased Ki-67 levels, is also associated with improved overall survival. This result suggests that EphA5 may modulate tumor proliferation and patient prognosis via a distinct biological mechanism, highlighting its potential as a favorable prognostic biomarker in EC.

Previous studies consistently highlighted the tumor-suppressive role of AMPK activation in EC. For example, metformin and the novel AMPK activator NT-1044 reportedly inhibit EC cell proliferation via AMPK-dependent suppression of the mTOR/S6K pathway and induction of apoptosis [29]. Furthermore, estrogen-induced upregulation of STC2 reportedly inhibits AMPK activity and promotes tumor growth, whereas STC2 knockdown restores AMPK phosphorylation and reduces EC cell viability [30]. Similarly, the transcription factor BHLHE40 reportedly enhances AMPK signaling by downregulating the phosphatase PPM1F, shifting tumor metabolism from glycolysis to oxidative phosphorylation [31]. Disruption of the GRP75–IP3R–Ca^2+^ axis triggered energy stress and AMPK activation in another study, thereby suppressing tumor cell proliferation [32], whereas a broader transcriptomic analysis demonstrated that AMPK activation modulates immune infiltration and downregulates oncogenic metabolic pathways in EC [33].

However, not all studies support a uniformly protective role for AMPK. Several investigations emphasized the context-dependent effects of AMPK activation. For instance, phosphorylation of INF2 by AMPK reportedly promotes mitochondrial fission and enhanced tumor cell adaptation to energy stress, thereby facilitating tumor progression in certain settings [34]. AMPK has also been described as a metabolic switch that, while typically tumor-suppressive, may promote survival under nutrient-limited conditions by inducing autophagy and metabolic flexibility [35]. In addition, glucose modulation studies revealed a dual role for AMPK: under low-glucose conditions, AMPK activation induces apoptosis and G1 arrest; conversely, under high-glucose conditions, AMPK activation co-occurs with enhanced proliferation [36].

Our findings highlight a unique context-dependent role of EphA5 in endometrial cancer, particularly in relation to proliferative signaling and metabolic regulation. The observed positive correlation between EphA5 and Ki-67 expression supports its link to tumor cell proliferation, which is consistent with the established role of Ki-67 as a marker of aggressive tumor biology and poor prognosis in EC. Notably, while high Ki-67 expression typically portends adverse outcomes, our data suggest that elevated EphA5 expression—despite its association with Ki-67—is linked to improved overall survival, implying a distinct, potentially modulatory role of EphA5 within proliferative signaling pathways. This paradox underscores the complexity of EphA5-mediated signaling and its potential as a favorable prognostic biomarker. Conversely, we observed an inverse association between EphA5 and pAMPK expression levels. Given the known tumor-suppressive functions of AMPK through the inhibition of mTOR signaling and induction of energy stress responses [37,38], the negative correlation between EphA5 and pAMPK suggests that EphA5 expression may be attenuated in metabolically restrained tumor environments. Taken together, these observations illustrate that the functional role of AMPK in EC is highly context-dependent. In alignment with this complexity, our study demonstrated that higher pAMPK expression levels were independently associated with lower EphA5 expression levels, whereas, paradoxically, high EphA5 expression levels correlated with better overall survival. This suggests that EphA5 may function as a compensatory or protective marker in the setting of reduced AMPK activity, thereby challenging the conventional view that AMPK activation universally confers favorable outcomes in EC. Furthermore, EPHA5 has been implicated in various malignancies, including cervical cancer, where integrative transcriptomic analyses identified it as part of oncogenic signaling networks that regulate proliferation, migration, and immune responses [24]. Collectively, these findings point to a multifaceted role of EphA5 in modulating tumor behavior across cancers, possibly through the intersection of proliferative and metabolic pathways, and suggest that its prognostic impact may vary according to tumor subtype and microenvironmental context.

In summary, our study revealed the complex interplay between EphA5, Ki-67, and AMPK signaling in EC. Although high Ki-67 expression levels were associated with increased EphA5 levels, consistent with its role in tumor proliferation, pAMPK expression was inversely correlated with EphA5 expression, suggesting that metabolic stress may suppress EphA5-mediated pathways. Notably, despite its positive correlation with proliferation markers, high EphA5 expression levels were predictive of better overall survival, highlighting the potential of EphA5 as a favorable prognostic biomarker, which went against our expectations. These results underscore the context-dependent roles of both AMPK and EphA5 in EC biology and suggest that EphA5 may serve as a protective factor in tumors with diminished AMPK activity.

### Limitations

Despite the statistically significant associations observed in our study, several limitations should be acknowledged. One of the most intriguing yet challenging findings was the paradoxical relationship between EphA5 expression and survival. Although high EhphA5 levels were positively associated with Ki-67 expression, a well-established marker of tumor proliferation, and inversely associated with pAMPK, a key energy-sensing tumor suppressor, they were nonetheless linked to improved overall survival. This unexpected outcome raises questions about the underlying biology of EphA5 and suggests that its role in endometrial cancer may be more complex than previously understood. It is possible that EphA5 contributes to tumor cell proliferation under certain conditions but also engages in protective or compensatory pathways that enhance long-term outcomes. Such context-dependent behavior is not uncommon in cancer biology, where signaling pathways often interact in nonlinear and stage-specific manners. Although our results are derived from a comprehensive analysis of human tumor tissues, they remain correlative in nature. To clarify the mechanistic role of EphA5, particularly its interplay with proliferative signaling and energy metabolism, future studies involving in vitro cellular models and in vivo animal experiments are essential. These functional investigations will be critical to determine whether EphA5 acts as a true driver of prognosis or merely reflects broader tumor microenvironmental dynamics. Furthermore, in order to obtain more accurate and reliable results, we specifically selected specimens that were pathologically confirmed as malignant tumors by pathologists and had not undergone any prior treatment. As a result, only 75 samples were collected, leading to a relatively small sample size. This limitation may have prevented us from clearly demonstrating certain trends.

## 5. Conclusions

In conclusion, our study identifies EphA5 as a context-dependent biomarker in endometrial cancer, demonstrating paradoxical associations with proliferative and metabolic markers. Although high EphA5 expression correlates with elevated Ki-67 levels and reduced pAMPK expression—features typically linked to tumor aggressiveness—it is independently associated with favorable overall survival outcomes. These findings suggest that EphA5 may play a compensatory or modulatory role in mitigating oncogenic stress, particularly in tumors with diminished AMPK activity. From a clinical perspective, EphA5 expression may serve as a valuable prognostic indicator to help stratify patients beyond traditional pathological parameters such as FIGO stage or histological grade. In particular, patients with high EphA5 expression could potentially benefit from less aggressive treatment approaches, provided that its protective role is further validated. Future translational research should explore EphA5 as a potential target or surrogate marker in precision oncology strategies for endometrial cancer.

## Figures and Tables

**Table 1:** Comparison of clinicopathological characteristics between low and high expression levels of EphA5 in EC.

Characteristic		Low Expression of EphA5 (n = 21)	High Expression of EphA5 (n = 54)	Total (n = 75)	*p*-Value
Age, years (median)		57.0 (52.0, 63.5)	54.0 (48.0, 60.0)	56.0 (50, 60)	0.096
Differentiation					0.014 *
	Well	2 (10%)	16 (30%)	18 (24%)	
	Moderate	11 (52%)	32 (59%)	43 (57%)	
	Poor	8 (38%)	6 (11%)	14 (19%)	
Size of tumor, cm (median)		4.0 (1.8, 6.5)	2.8 (1.5, 5.0)	3.0 (1.5, 5.0)	0.282
FIGO stage					0.006 **
	I	13 (62%)	48 (89%)	61 (81%)	
	II	2 (10%)	4 (7%)	6 (8%)	
	III	6 (29%)	2 (4%)	8 (11%)	

Values are displayed as median (quartile 1, quartile 3) or n (%). * *p* < 0.05, ** *p* < 0.01. FIGO, International Federation of Gynecology and Obstetrics.

**Table 2:** Comparison of tumor biomarker expression (H-score) between low and high expression levels of EphA5 in EC.

Biomarker	Low Expression of EphA5 (Median)	High Expression of EphA5 (Median)	Total	*p*-Value
Caspase-3	6.3 (4.2, 9.4)	8.1 (5.3, 13.6)	7.1 (5.2, 12.8)	0.075
Ki-67	1.8 (1.2, 6.3)	6.2 (1.2, 22.1)	5.2 (1.3, 16.1)	0.050
CD31	6.4 (4.8, 8.6)	7.1 (5.4, 11.8)	6.9 (5.1, 96)	0.322
E-cadherin	107.8 (102.3, 113.7)	110.1 (104.3, 116.2)	109.4 (103.5, 116.1)	0.351
N-cadherin	2.8 (1.7, 7.5)	10.8 (4.6, 39.5)	7.6 (2.5, 34.1)	0.003 **
Fibronectin	16.2 (3.2, 34.5)	5.7 (1.9, 32.9)	8.9 (2.1, 31.9)	0.302
pAkt	1.7 (1.0, 3.8)	3.4 (2.1, 7.1)	3.0 (1.6, 6.5)	0.003 **
pErk	0.2 (0.1, 3.3)	0.6 (0.1, 4.6)	0.4 (0.1, 4.6)	0.173
pStat3	0.1 (0.0, 0.1)	0.2 (0.1, 0.5)	0.1 (0.1, 0.4)	0.001 **
pAMPK	22.6 (14.8, 27.7)	13.5 (10.1, 19.8)	14.8 (12.0, 22.6)	0.004 **

Values are displayed as median (quartile 1, quartile 3). ** *p* < 0.01.

**Table 3:** Univariate and multivariable logistic regression analysis of factors associated with a high EphA5 expression level in EC.

Factors		Univariate	*p*-Value	Multivariable	*p*-Value
Age		0.95 (0.90–1.01)	0.090	0.96 (0.87–1.05)	0.370
Differentiation					
	Well	Reference		Reference	
	Moderate	0.36 (0.07–1.84)	0.221	0.14 (0.02–1.17)	0.070
	Poor	0.09 (0.02–0.57)	0.010 *	0.06 (0.00–1.05)	0.054
Size of tumor		0.90 (0.76–1.06)	0.210		
Stage					
	I	Reference		Reference	
	II	0.54 (0.09–3.29)	0.505	0.67 (0.05–6.63)	0.671
	III	0.09 (0.02–0.50)	0.006 **	0.06 (0.00–0.95)	0.046 *
Markers					
	Caspase-3	1.10 (0.99–1.21)	0.076		
	Ki-67	1.07 (1.00–1.13)	0.039 *	1.08 (1.01–1.16)	0.024 *
	CD31	1.05 (0.96–1.15)	0.280		
	E-cadherin	1.02 (0.96–1.09)	0.492		
	N-cadherin	1.02 (1.00–1.05)	0.082		
	Fibronectin	0.99 (0.98–1.01)	0.320		
	pAkt	1.16 (0.98–1.37)	0.094		
	pErk	1.01 (0.96–1.06)	0.615		
	pStat3	99.83 (1.59–6287.31)	0.029 *	55.04 (0.30–10,038.08)	0.131
	pAMPK	0.92 (0.87–0.99)	0.016 *	0.89 (0.81–0.98)	0.024 *

Values are displayed as odds ratios (95% confidence intervals). * *p* < 0.05, ** *p* < 0.01.

**Table 4:** Pearson correlation analysis between EphA5 expression levels and patient demographics and biomarkers, stratified according to cancer stage.

	Stage I (n = 61)		Stage II (n = 6)		Stage III (n = 8)	
Factors	Pearson Correlation	*p*-Value	Pearson Correlation	*p*-Value	Pearson Correlation	*p*-Value
Age	−0.192	0.139	0.112	0.832	0.298	0.473
Grading	−0.262	0.041 *	0.500	0.312	0.218	0.604
Size of tumor	0.073	0.578	0.006	0.990	−0.419	0.301
Caspase-3	0.177	0.171	0.389	0.446	0.571	0.139
Ki-67	0.255	0.047 *	−0.471	0.345	0.909	0.002 **
CD31	0.128	0.325	0.339	0.511	0.445	0.269
E-cadherin	−0.034	0.793	−0.234	0.656	0.486	0.222
N-cadherin	0.121	0.353	0.047	0.930	0.882	0.004 **
Fibronectin	−0.004	0.974	−0.140	0.792	−0.412	0.310
pAkt	0.219	0.089	0.480	0.335	0.118	0.781
pErk	−0.037	0.776	0.020	0.970	0.652	0.080
pStat3	0.169	0.194	0.672	0.144	0.524	0.182
pAMPK	−0.447	<0.001 ***	−0.008	0.988	−0.029	0.946

* *p* < 0.05, ** *p* < 0.01, and *** *p* < 0.001.

## Data Availability

The raw data supporting the conclusions of this article will be made available by the authors on request.

## Supplementary Materials

The following supporting information can be downloaded at: https://www.mdpi.com/article/10.3390/jcm14155360/s1, Table S1: Expression differences of tumor biomarkers by EphA5 status in each FIGO stage. (Median (Q1, Q3)); Table S2: Univariate and multivariable logistic regression analyses of factors associated with high EphA5 expression levels in stage I endometrial cancer; Table S3: Univariate logistic regression analysis of factors associated with high EphA5 expression levels in stages II and III endometrial cancer.

## Abbreviations

The following abbreviations are used in this manuscript:

aOR	Adjusted odds ratio
CI	Confidence interval
EC	Endometrial cancer
EphA5	Eph receptor A5
FIGO	International Federation of Gynecology and Obstetrics
HG-ESS	High-grade endometrial stromal sarcoma
LG-ESS	Low-grade endometrial stromal sarcoma
UUS	Undifferentiated uterine sarcoma
