# Supplementary material for: EphA5 Expression Predicts Better Survival Despite an Association with Proliferative Activity in Endometrial Cancer
Source: J Clin Med. 2025 Jul 29;14(15):5360. doi: 10.3390/jcm14155360 (PMC12347129; doi:10.3390/jcm14155360)
Supplement: Supplementary file 1 [file jcm-14-05360-s001.zip › jcm-3730419-supplementary.pdf]

Table S1. Expression differences of tumor biomarkers by EphA5 status in each FIGO stage. (Median (Q1, Q3))

	FIGO Stage I				FIGO Stage II				FIGO Stage III			
**Biomarker**	**Low**	**High**	**Total**	**p-value**	**Low#**	**High**	**Total**	**p-value**	**Low**	**High#**	**Total**	**p-value**
**Caspase-3**	6.3 (5.1, 7.3)	7.2 (5.2, 13.1)	6.8 (5.2, 11.4)	0.214	(2.9, 8.9)	13.9 (8.0, 21.1)	11.7 (6.6, 19.8)	0.533	7.0 (2.3, 12.3)	(12.8, 13.5)	10.4 (3.1, 13.3)	0.143
**Ki-67**	1.5 (1.2, 6.3)	5.5 (1.4, 20.7)	5.3 (1.3, 15.0)	0.067	(14.9, 23.5)	10.6 (0.1, 23.9)	17.9 (0.2, 26.7)	0.533	1.9 (0.9, 3.5)	(18.1, 28.0)	2.6 (1.3, 14.8)	0.071
**CD31**	6.4 (5.0, 7.5)	6.9 (5.2, 9.4)	6.8 (5.2, 9.2)	0.459	(3.5, 6.5)	8.9 (4.7, 18.0)	7.5 (4.2, 14.2)	0.533	6.7 (4.0, 15.5)	(11.7, 17.6)	9.7 (4.9, 20.8)	0.429
**E-cadherin**	108.3 (104.4, 120.1)	110.6 (103.7, 117.0)	110.2 (103.9, 117.7)	0.916	(104.8, 110.6)	108.9 (107.3, 110.2)	108.9 (106.4, 111.9)	1.000	101.5 (100.4, 108.9)	(106.8, 108.4)	104.5 (100.6, 109.6)	0.429
**N-cadherin**	2.8 (2.1, 21.6)	10.5 (4.1, 39.1)	9.0 (2.8, 36.1)	0.051	(0.3, 17.7)	8.7 (2.4, 48.4)	8.7 (0.9, 41.5)	1.000	2.7 (1.0, 4.4)	(24.4, 42.4)	3.9 (1.3, 19.7)	0.071
**Fibronectin**	16.2 (3.2, 26.0)	5.7 (1.9, 34.9)	8.9 (2.0, 31.6)	0.585	(1.0, 50.2)	11.3 (3.7, 92.3)	11.3 (2.4, 103.9)	1.000	19.0 (4.9, 96.5)	(1.1, 1.8)	8.9 (1.5, 74.9)	0.286
**pAkt**	1.7 (1.1, 3.2)	3.2 (2.2, 6.9)	2.7 (1.8, 6.2)	0.006**	(1.0, 1.0)	6.1 (1.2, 17.1)	1.2 (1.04, 12.9)	0.133	3.4 (0.8, 7.6)	(6.5, 6.5)	3.8 (1.4, 6.5)	0.286
**pErk**	0.3 (0.1, 10.1)	0.6 (0.2, 4.7)	0.6 (0.1, 5.9)	0.616	(0.2, 0.4)	0.4 (0.0, 0.9)	0.4 (0.1, 0.8)	1.000	0.2 (0.0, 0.4)	(0.1, 14.5)	0.2 (0.1, 0.5)	0.643
**pStat3**	0.1 (0.0, 0.1)	0.2 (0.1, 0.4)	0.1 (0.1, 0.4)	0.012	(0.0, 0.0)	0.4 (0.1, 0.5)	0.2 (0.1, 0.8)	0.267	0.1 (0.0, 0.3)	(0.2, 0.3)	0.2 (0.0, 0.4)	0.286
**pAMPK**	25.9 (19.8, 28.9)	13.6 (9.9, 19.6)	14.8 (11.7, 23.1)	<0.001***	(19.1, 19.1)	20.4 (7.5, 29.1)	19.1 (11.5, 27.9)	1.000	14.8 (7.5, 18.3)	(13.1, 13.1)	13.9 (10.1, 16.4)	0.643

**$P < 0.01$, *** $P < 0.001$

Owing to the small sample size, the expression levels are reported as ranges (minimum to maximum) rather than using median or interquartile statistics to avoid overinterpretation.

Table S2. Univariate and multivariable logistic regression analyses of factors associated with high EphA5 expression levels in stage I endometrial cancer.

	Univariate	p-value	Multivariable	p-value
Age	0.95 (0.90-1.02)	0.146	0.98 (0.89-1.08)	0.703
Differentiation				
Well	Reference		Reference	
Moderate	0.47 (0.09-2.48)	0.372	0.19 (0.02-2.12)	0.177
Poor	0.08 (0.01-0.84)	0.035*	0.01 (0.00-0.22)	0.006**
Size	1.09 (0.80-1.49)	0.572		
Marker				
Caspase-3	1.09 (0.96-1.24)	0.188		
Ki-67	1.10 (0.99-1.21)	0.075		
CD31	1.07 (0.93-1.22)	0.336		
E-cadherin	0.99 (0.92-1.06)	0.789		
N-cad	1.01 (0.99-1.04)	0.353		
Fibronectin	1.00 (0.98-1.02)	0.973		
pAkt	1.37 (0.95-1.97)	0.089		
pErk	0.99 (0.95-1.04)	0.772		
pStat3	79.87 (0.37-17086.71)	0.110		
pAMPK	0.87 (0.80-0.95)	0.002**	0.81 (0.72-0.92)	0.001**

*p < 0.05, **P < 0.01

Table S3. Univariate logistic regression analysis of factors associated with high EphA5 expression levels in stages II and III endometrial cancer.

	Stage II (n=6)		Stage III (n=8)	
	Univariate	p-value	Univariate	p-value
Age	1.04 (0.79-1.38)	0.784	1.10 (0.88-1.37)	0.417
Differentiation				
Well	Reference		Reference	
Moderate	1615474843 (0.00-)	0.999	646189766 (0.00-)	>0.999
Poor	N/A	N/A	N/A	N/A
Size	1.00 (0.64-1.58)	0.988	0.71 (0.38-1.32)	0.280
Marker				
Caspase-3	1.15 (0.85-1.56)	0.370	N/A	N/A
Ki-67	0.90 (0.74-1.10)	0.306	1.14 (0.91-1.43)	0.245
CD31	1.18 (0.77-1.81)	0.445	1.40 (0.80-2.44)	0.240
E-cadherin	0.87 (0.54-1.41)	0.577	N/A	N/A
N-cadherin	1.01 (0.93-1.09)	0.909	0.63 (0.23-1.68)	0.352
Fibronectin	0.99 (0.96-1.03)	0.734	1.05 (0.79-1.40)	0.742
pAkt	3.508E+127 (0.00-)	0.980	1.31 (0.36-4.72)	0.683
pErk	1.12 (0.01-127.67)	0.962	2072.53 (0.02-184613689.9)	0.189
pStat3	6,311,802.48 (0.00-2.043E+22)	0.390	79.87 (0.37-17086.71)	0.110
pAMPK	1.00 (0.81-1.23)	0.985	0.99 (0.74-1.33)	0.936

N/A, not applicable
